# Bite of a Copperhead—“Trigonocephalus Contortix”—Treated with Whisky

**Published:** 1853-04

**Authors:** 


### *Bite of a Copperhead—" Trigonocephalus Contortix"—treated with Whisky.* By N. Harris Morange, M.D., of Abbeville, S. C.

On the 21st of June last, I was called to see a negro man belonging to Capt. P——, of Abbeville district. Found him partially delirious; skin hot and dry; pulse very much excited, ranging from 100 to 120; left leg and ankle swollen to a great degree. Upon making inquiry into the history of this case, I learned that the patient had been bitten about 12 hours previously by a "trigonocephalus," or, as it is frequently styled in this part of the country, copperhead or highland mockeson. This very poisonous reptile was concealed beneath the step of a *meat-house*, and inflicted a wound upon the inside of the foot, near the ankle joint. I immediately applied a ligature above the seat of affection—prescribed poultices over the wound; and olive oil, ammonia, &c., internally.

22d. The patient is *in statu quo*—no abatement of the swelling, delirious: ordered whisky, *ad libitum.*

23d. No decided improvement—still anxious, restless, and uneasy; skin hot and dry. Continued the whisky, combined with capsicum: it was administered, until the patient was fully under its influence, without regard to quantity. Left opium to be given if necessary.

24th. Had passed the "crisis." A profuse perspiration was over his entire system; the tumefaction was subsiding; the delirium had ceased; he spoke rationally, and speedily convalesced.

Gibson says—"Of the numerous American serpents, two species only are known to be poisonous—the crotalus or rattlesnake and copperhead." If he includes under the common name of copperhead, both the highland and the water-mockeson, then we concur with him in the assertion. The two latter are of the same family, but not of the same species, which is abundantly manifest by their mode of living.

The same writer says: "These reptiles are more lively, and their venom more active during the very warm weather. Upon the approach of the cold season, they become languid, and then strike reluctantly, and frequently without any ill consequence."

The interesting case which I witnessed whilst a student in the University of New York, furnishes a striking proof of the speedy operation of the poison even in the dead of winter.

Dr. W., of that city, was bitten on the hand, by a rattlesnake, sent to him by a friend from the State of Alabama. The hand soon began to swell, and in a few hours the whole arm was very much tumified, presenting a mottled appearance, even to the shoulder and axilla. He had the best medical advice the city afforded; yet, after intense suffering for two or three days, he died.

I have no doubt, that if the *alcoholic* treatment had been instituted in this case, with the application of the ligature above the seat of affection, (which last was timely suggested by some of the Southern students present,) Dr. W. would have recovered.—*Southern Med. and Surg. Journal.*

